# Trigger mechanisms of secondary sclerosing cholangitis in critically ill patients

**DOI:** 10.1186/s13054-015-0861-5

**Published:** 2015-03-31

**Authors:** Silke Leonhardt, Wilfried Veltzke-Schlieker, Andreas Adler, Eckart Schott, Roland Hetzer, Walter Schaffartzik, Michael Tryba, Peter Neuhaus, Daniel Seehofer

**Affiliations:** Department of General, Visceral and Transplantation Surgery, Charité-Universitätsmedizin Berlin, Augustenburger Platz 1, Berlin, 13353 Germany; Clinic for Gastroenterology, Hepatology and Infectiology, Heinrich Heine University, Moorenstrasse 5, Düsseldorf, 40225 Germany; Endoscopy Unit, Department of Gastroenterology and Hepatology, Endocrinology, Diabetes and Metabolic Diseases, Charité- Universitätsmedizin Berlin, Augustenburger Platz 1, Berlin, 13353 Germany; Department of Cardiothoracic and Vascular Surgery, Deutsches Herzzentrum Berlin, Augustenburger Platz 1, Berlin, 13353 Germany; Department of Anaesthesiology, Intensive Care Medicine and Pain Therapy, Unfallkrankenhaus Berlin, Warener Strasse 7, Berlin, 12638 Germany; Department of Anaesthesiology, Intensive Care Medicine and Pain Therapy, Klinikum Kassel, Mönchebergstrasse 41, Kassel, 34125 Germany

## Abstract

**Introduction:**

In recent years the development of secondary sclerosing cholangitis in critically ill patients (SSC-CIP) has increasingly been perceived as a separate disease entity. About possible trigger mechanisms of SSC-CIP has been speculated, systematic investigations on this issue are still lacking. The purpose of this study was to evaluate the prevalence and influence of promoting factors.

**Methods:**

Temporality, consistency and biological plausibility are essential prerequisites for causality. In this study, we investigated the temporality and consistency of possible triggers of SSC-CIP in a large case series. Biological plausibility of the individual triggers is discussed in a scientific context. SSC-CIP cases were recruited retrospectively from 2633 patients who underwent or were scheduled for liver transplantation at the University Hospital Charité, Berlin. All patients who developed secondary sclerosing cholangitis in association with intensive care treatment were included. Possible trigger factors during the course of the initial intensive care treatment were recorded.

**Results:**

Sixteen patients (68% males, mean age 45.87 ± 14.64 years) with a confirmed diagnosis of SSC-CIP were identified. Of the 19 risk factors investigated, particularly severe hypotension with a prolonged decrease in mean arterial blood pressure (MAP) to <65 mmHg and systemic inflammatory response syndrome (SIRS) were established as possible triggers of SSC-CIP. The occurrence of severe hypotension appears to be the first and most significant step in the pathogenesis. It seems that severe hypotension has a critical effect on the blood supply of bile ducts when it occurs together with additional microcirculatory disturbances.

**Conclusions:**

In critically ill patients with newly acquired cholestasis the differential diagnosis of SSC-CIP should be considered when they have had an episode of haemodynamic instability with a prolonged decrease in MAP, initial need for large amounts of blood transfusions or colloids, and early development of a SIRS.

## Introduction

Medical and technical progress in critical care medicine has increased survival rates of patients with life-threatening injuries. However, as critically ill patients survive more often, new ICU-acquired diseases have emerged. Recent years have seen increasing reports of a new entity of ICU-associated diseases. The development of a secondary sclerosing cholangitis in critically ill patients (SSC-CIP) was first observed in patients suffering burns or polytrauma, but subsequently also following other acute life-threatening events [1-4]. SSC-CIP is characterized by cholestasis and bile duct necrosis, and can be diagnosed on the basis of typical cholangiographic findings. It appears to be irreversible and progresses rapidly to biliary cirrhosis and liver failure. Without exception, the medical history of all reported patients with SSC-CIP revealed previous intensive care treatment [1-4]. There is no doubt that the triggers of SSC-CIP are to be found in the time window between the life-threatening initial event and the occurrence of cholestasis. Whether the disease is caused by the initial injury itself or by the intensive care treatment is not yet clear. Therapeutic options for SSC-CIP are limited, the mortality rate during initial ICU stay is high and many surviving patients have to undergo liver transplantation. If ICU-associated causes could be identified, appropriate avoidance strategies might help prevent the development of SSC-CIP. Currently available reports speculate on a range of possible causes of SSC-CIP – such as sepsis, high-dose catecholamines and ischemia [2,3]. However, specific studies are lacking. Up to now, no studies have examined which of the suspected triggers can, in fact, be verified in patients with SSC-CIP. The aim of the present study was to identify and evaluate possible trigger factors of SSC-CIP.

## Material and methods

Records of patients who received (2,473) or were listed for (160) orthotopic liver transplantation (OLT), at the Charité University Hospital Berlin between 1 January 1990 and 8 May 2011 were carefully reviewed. A total of 16 patients with a confirmed diagnosis of SSC-CIP were identified. The diagnosis of SSC-CIP was based on typical findings on endoscopic retrograde cholangiography (ERC), such as the presence of casts or destruction of intrahepatic bile duct branches. Other causes of cholestasis were excluded by comprehensive differential diagnostic examinations. Additionally, liver histology was available in 11 patients. At the time of data collection, 9 of the 16 patients had already been transplanted, 5 were still on the waiting list, and 2 had died while still on the waiting list. As tertiary care centres the Departments of Transplantation Surgery and Gastroenterology receive patients for liver transplantation as well as for endoscopic or percutaneous biliary interventions from various other hospitals. Therefore, most of our patients underwent initial intensive care treatment at outside hospitals. The records of these patients’ initial hospital treatment elsewhere were reviewed in detail for specific risk factors including: presence and duration of severe hypotension, total parenteral nutrition (TPN), number of red blood cell (RBC) transfusions, use of colloidal solutions, systemic inflammatory response syndrome (SIRS)/sepsis, antibiotic treatment, positive cultures prior to cholestasis, number of days of mechanical ventilation prior to the development of cholestasis, total duration of mechanical ventilation, ventilation strategies with positive end-expiratory pressure (PEEP) >10 cm H_2_O, a paO_2_/FiO_2_ < 150 mmHg, use of vasopressors/inotropes (epinephrine, norepinephrine, dopamine, dobutamine), anaesthetic agents (propofol, midazolam, ketamine, sufentanil) and signs of tissue damage. The list included all risk factors capable of destroying the bile duct directly or indirectly. Only those risk factors presenting prior to the onset of cholestasis were recorded. The occurrence of cholestasis was interpreted as indicative of incipient bile duct destruction. The onset of cholestasis was defined as an increase in cholestatic parameters (gamma-glutamyl transpeptidase (GGT), alkaline phosphatase (ALP), bilirubin) to more than twice the upper normal limit. Severe hypotension was defined as a sustained (≥60 minutes) mean arterial blood pressure (MAP) of less than 65 mmHg requiring fluid and/or catecholamine administration. All data are presented as means ± standard deviations of the mean. For all vasopressors/inotropes administered, both the mean doses and the peak doses were recorded. Mean dose: calculated from total amount administered prior to cholestasis onset, administration time prior to cholestasis and body weight (expressed in μg · kg^−1^ · min^−1^). Peak dose: transitory maximum dose in μg · kg^−1^ · min^−1^. Doses were defined as low dose <0.05 μg · kg^−1^ · min^−1^, medium dose = 0.05 to 0.2 or high dose >0.2 μg · kg^−1^ · min^−1^.

The study was approved by the local ethics committee of the Charité, and all surviving patients gave their written informed consent to having their data recorded.

## Results

The mean age of the patients (eleven men and five women) at the time of diagnosis was 46 ± 14.6 years (median 48 years, range 18 to 63). All 16 patients had a history of intensive care treatment necessitated by a prior life-threatening event. A summary of the life-threatening events/causes for admission to ICU and demographic data are shown in Table 1. None of the patients had a previous history of liver or bile duct diseases, and had unremarkable liver function tests on admission to ICU. During intensive care treatment, all patients developed elevated liver enzymes of a cholestatic pattern. In 14 of the patients, liver enzymes were checked regularly during the first 10 days, and cholestasis was diagnosed after a mean of 7.86 ± 2.98 days (median seven days). In two patients, liver enzymes were first determined on day 12 and 18, respectively.

**Table 1:** **Demographic characteristics**

**Characteristic**	**Number**
Total number of cases	16
Gender (male/female)	11/5
Mean age at diagnosis (years) ± SDM	45.87 ± 14.64
Median age at diagnosis (years), range	48 (18 to 63)
Mean follow-up (months) ± SDM	62.7 ± 32
Median follow-up (months), range	62 (5.6 to 171)
**Initial event**	
Polytrauma	7
Burn injury	1
Cardiac surgery	4
Severe postoperative bleeding after gynaecologic surgery	1
Subarachnoid haemorrhage	1
Respiratory insufficiency due to acute exacerbation of COPD	2
**Duration of intensive care treatment**	
Mean (d) ± SDM	38.75 ± 23.87
Median (d), range	31 (9 to 82)
**Duration of mechanical ventilation**	
Mean (d) ± SDM	25.5 ± 14.3
Median (d), range	21.5 (8 to 53)

COPD, chronic obstructive pulmonary disease; SDM, standard deviation of the mean.

**Figure 1:**
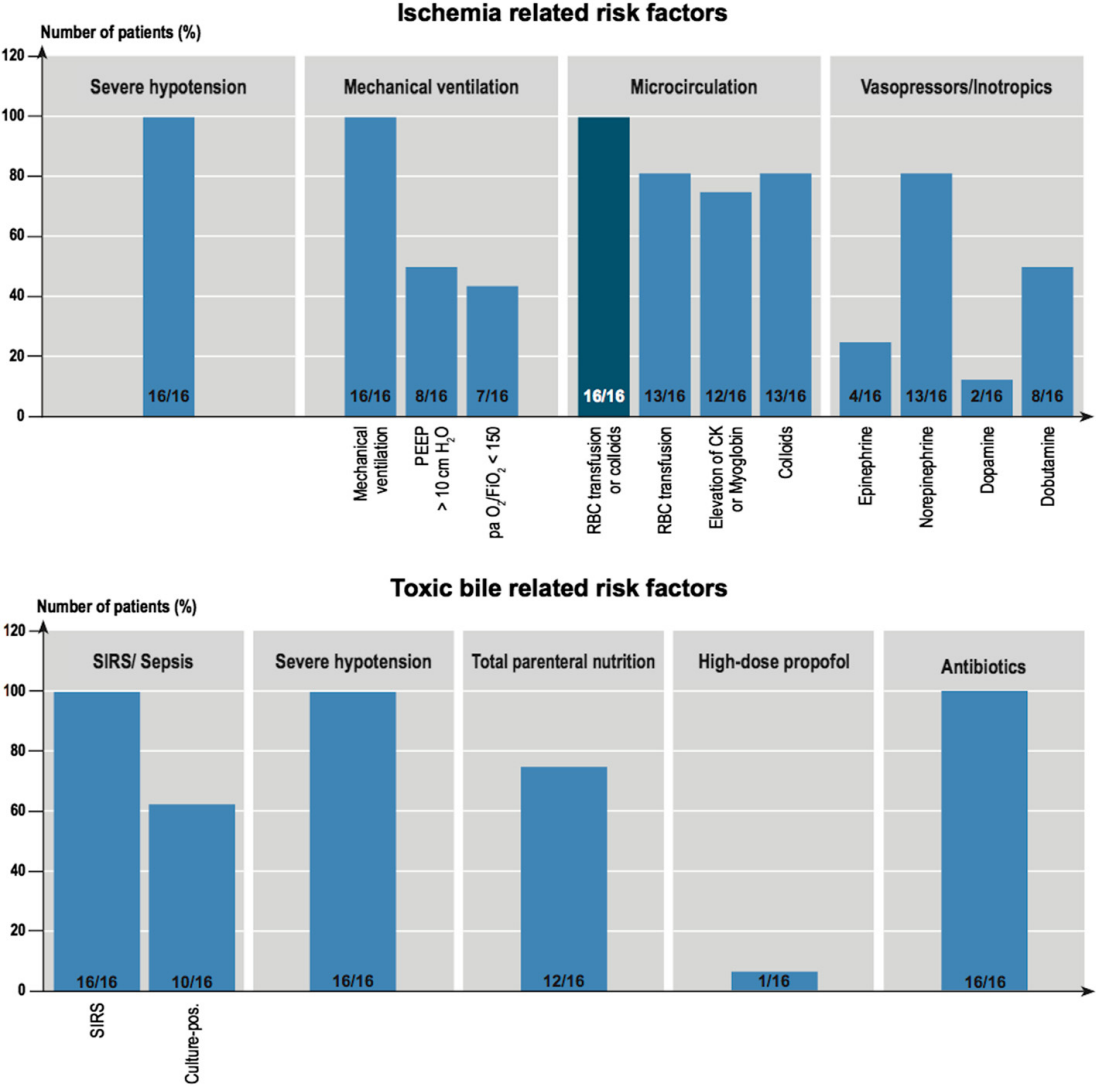
**Ischemia and toxic bile-related risk factors.** CK, creatine kinase; PEEP, positive end–expiratory pressure; RBC, red blood cells; SIRS, systemic inflammatory response syndrome.

**Figure 2:**
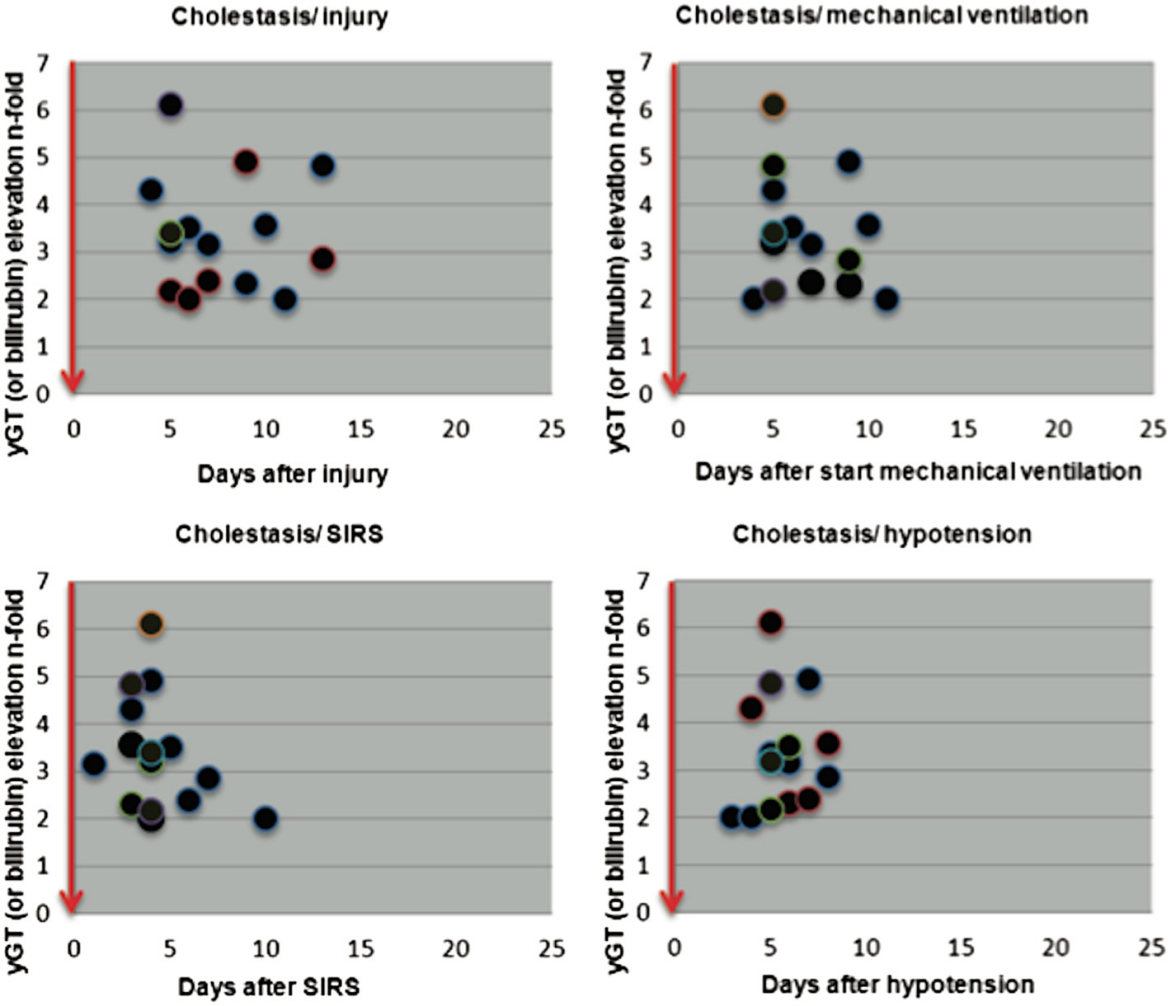
**Onset of cholestasis following impact of several risk factors.** (initial injury, mechanical ventilation, severe hypotension, SIRS). SIRS, systemic inflammatory response syndrome.

**Figure 3:**
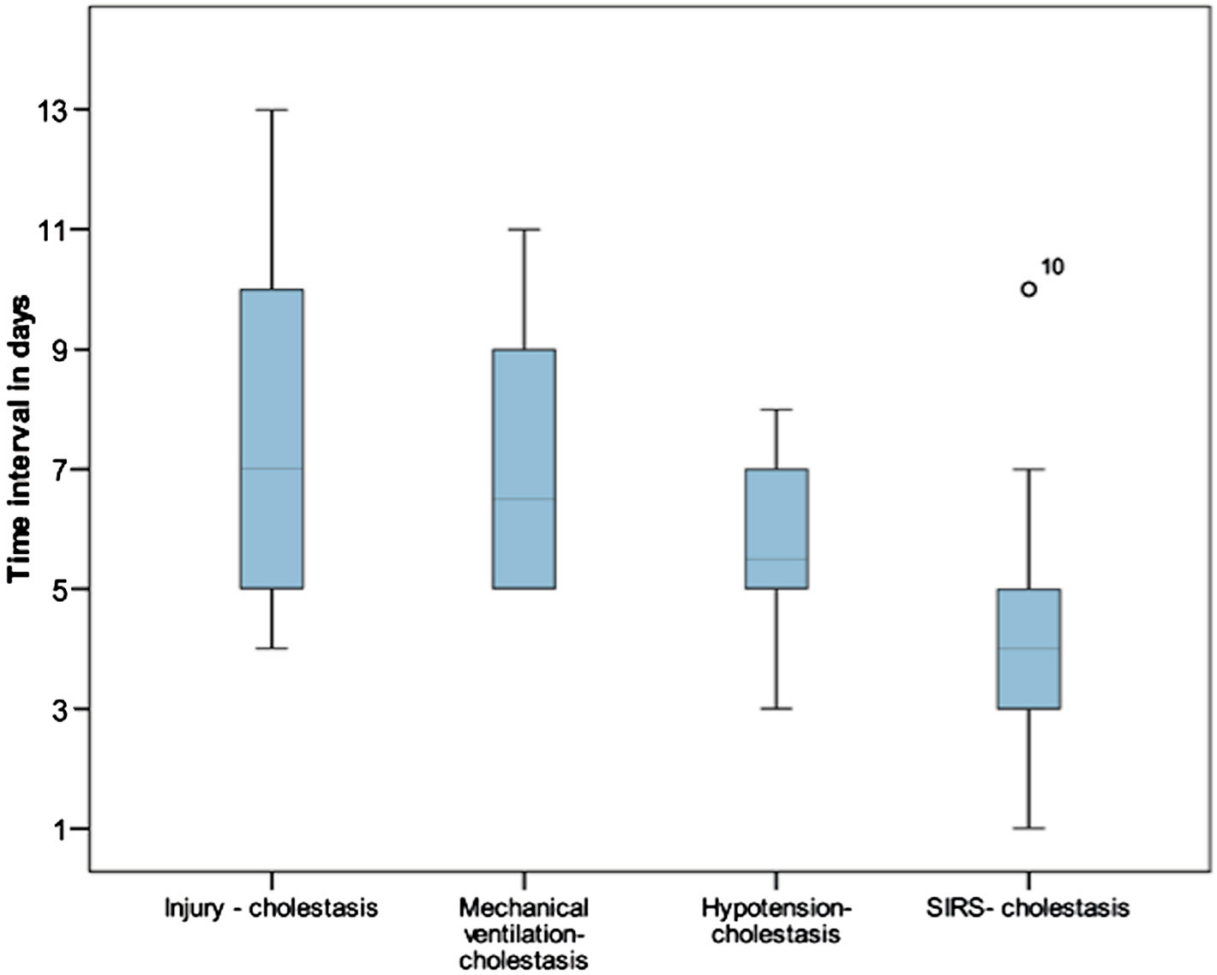
**Boxplot –Time interval between several risk factors (initial injury, mechanical ventilation, severe hypotension, SIRS) and onset of cholestasis.** SIRS, systemic inflammatory response syndrome.

**Figure 4:**
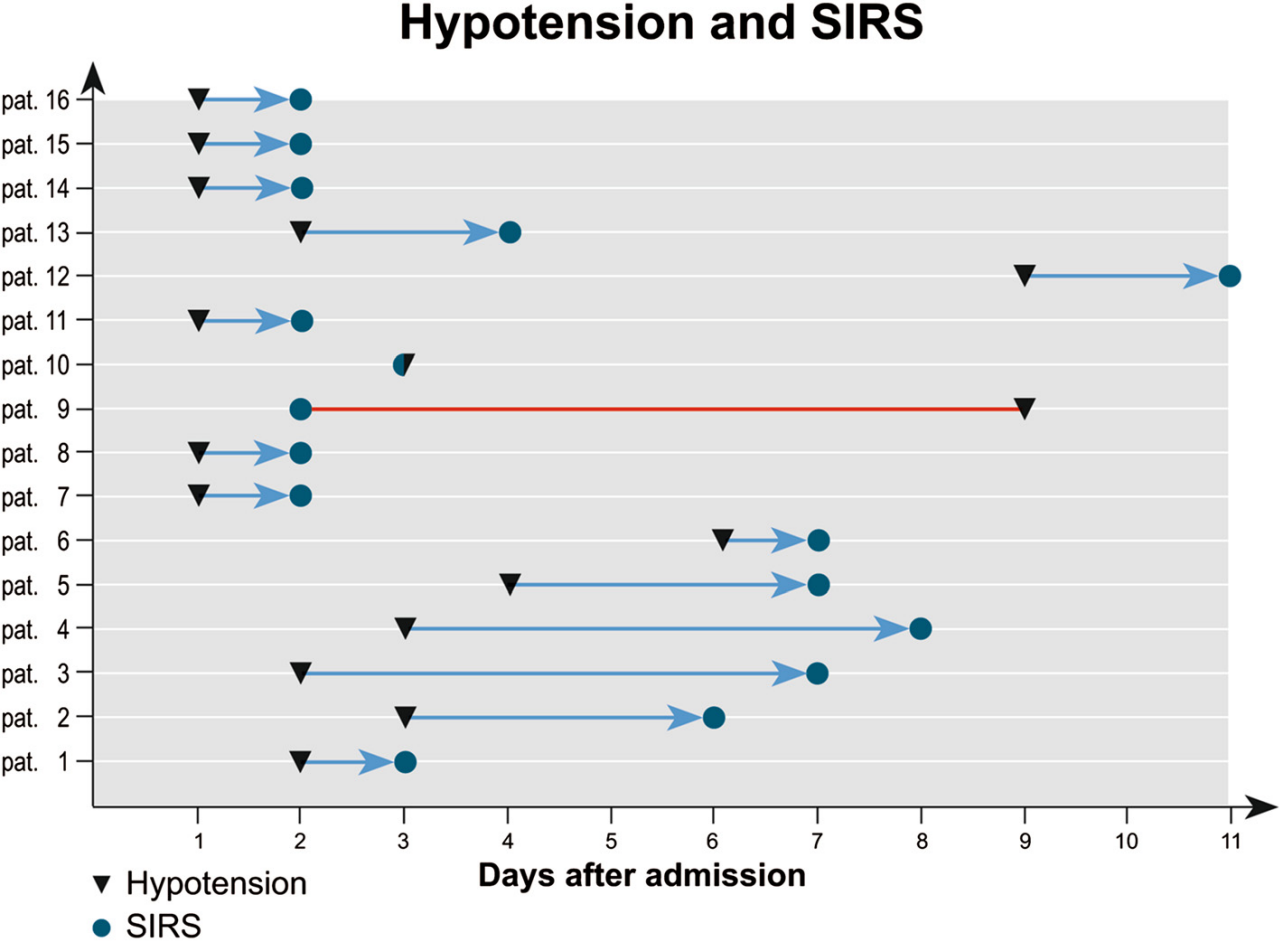
**Chronological sequence of severe hypotension and SIRS.** SIRS, systemic inflammatory response syndrome.

The data regarding the 19 trigger factors investigated are summarised in Figure 1 and Table 2. Between the initial event and the onset of cholestasis all patients experienced an episode of severe haemodynamic instability with a decrease in MAP to below 65 mm Hg lasting for at least 60 minutes, often more than 120 minutes. A later occurrence of this haemodynamic instability after the initial event correlated with a later increase in cholestasis parameters. The onset of cholestasis was most closely time-related to the risk factor ‘severe hypotension’ and the interval between hypotension and cholestasis showed the smallest standard deviation (1.44). The onset of cholestasis correlated less closely with the initiation of ventilation or the onset of SIRS, and least closely with the initial life-threatening event (Figures 2 and 3). Severe hypotension cannot be considered as a consequence of sepsis, since 14/16 patients had the episode of haemodynamic instability some days prior to the onset of SIRS/sepsis (1.8 ± 1.2 days) (Figure 4).

**Table 2:** **Risk factors (prior to cholestasis) in 16 cases with SSC-CIP**

**No**	**Gender**	**Duration ICU (d)**	**MAP <65 mmHg**	**Ventilation**	**PaO**_**2**_ **/FiO**_**2**_ **< 150**	**PEEP >10 cmH**_**2**_ **0**	**Transfusion units RBC**	**Colloidal solutions**		**Signs of tissue damage**		**SIRS**	**Pos. Cultures**		**TPN/ for d**	**High dose Propofol**	**AB**
								**HES 500 ml**	**GF 500 ml**	**CK (U/l)**	**Myoglobin (μg/l)**		**BC**	**Other Isolates**			
**1**	F	28	+	+	-	+12.0	18	7x	3x	n.a.	.n.a.	+	-	-	+/4	-	+
**2**	M	24	+	+	+	+11.0	12	-	-	5323	n.a.	+	-	Tr+	+/5	-	+
**3**	M	23	+	+	-	+12.0	20	4x	-	2348	3169	+	-	Tr+	+5	-	+
**4**	M	62	+	+	+	+10.0	4	8x	-	4920	2759	+	-	Tr+	+/6	+	+
**5**	M	70	+	+	-	+12.6	4	-	8x	4026	n.a.	+	-	-	+/6	-	+
**6**	M	48	+	+	+	+14.0	2	3x	2x	n.a.	n.a.	+	-	-	+/8	-	+
**7**	M	15	+	+	-	-	0	4x	-	n.a.	n.a.	+	-	Tr+	-	-	+
**8**	M	77	+	+	-	-	16	-	-	1244	n.a.	+	-	Tr+	-	-	+
**9**	M	82	+	+	-	-	26	1x	-	3645	29746	+	+	Ws+	+/2	-	+
**10**	F	54	+	+	+	+10.4	0	4x	-	536	n.a.	+	-	VC/U+	+/13	-	+
**11**	F	25	+	+	-	-	19	5x	-	345	n.a.	+	-	-	-	-	+
**12**	M	34	+	+	+	+14.0	10	-	1x	7250	7084	+	-	-	+/5	-	+
**13**	F	39	+	+	+	-	0	2x	-	1212	n.a.	+	-	Tr+	+/4	-	+
**14**	M	17	+	+	+	-	10	-	-	4975	n.a.	+	-	Tr+	-	-	+
**15**	M	13	+	+	-	-	4	1x	-	677	n.a.	+	-	Tr+	+/3	-	+
**16**	F	9	+	+	-	-	7	9x	1x	3166	n.a.	+	-	-	+/3	-	+

AB, antibiotics; BC, blood culture; CK, creatine kinase (normal range <170 U/l); d, days; GF, gelafusine; HES, hydroxyethyl starch; ICU, intensive care unit; MAP, mean arterial pressure; n.a., data not available; PEEP, positive end–expiratory pressure; RBC, red blood cells;, myoglobin (normal range <76 μg/l); SIRS, systemic inflammatory response syndrome; TPN, total parenteral nutrition/for n days; Tr, tracheal secretion; U, urine; VC, venous catheter tip; Ws, Wound swab.

All patients needed vasopressors and/or inotropes to stabilise the circulation. The doses required are shown in Table 3. In total, 81% of the patients received norepinephrine and 25% epinephrine. Between hospital admission and cholestasis onset 13 patients required transfusions (mean 11.7 ± 7.55 units; range 2 to 26; median 10) and 81% required colloidal solutions (hydroxyethyl starch 6% or 10%; Gelafusine 4%) to stabilise the circulatory system. Taken together, all 16 patients received either RBCs or colloids to support systemic perfusion. Prior to the onset of cholestasis all 16 patients underwent mechanical ventilation. The duration of ventilation prior to cholestasis was a mean 7.4 ± 2.4 days (median 7 days). Ventilation strategies with a PEEP >10 cm H_2_O were applied in 8/16 patients. A PaO_2_/FiO_2_ ratio <150 mm Hg persisting for several hours was observed in 7/16 patients. Laboratory investigations revealed signs of tissue damage (elevated creatine kinase or myoglobin) in 12 of 16 patients. According to the criteria of the Consensus Conference Committee of the American College of Chest Physicians/Society of Critical Care Medicine [5] all 16 patients (100%) met the SIRS criteria within the period between initial injury and cholestasis onset, specifically all 16 patients met at least two criteria and 6 patients met three criteria. During that time period, only one patient had positive blood cultures, 8/16 patients had pathogens in the tracheal secretion, 1/16 patient had a positive wound swab, and 1/16 patient had pathogens in the urine and on the tip of the central venous catheter. The most common microorganism isolated was *Escherichia coli*, which was found in three patients. All 16 patients received antibiotic treatment, which was initiated as calculated antibiosis. Ten different classes of antibiotics and 19 different single substances were applied. Prior to the onset of cholestasis, changes of therapy were made in 13 patients due to lack of clinical response. The majority of patients received two different antibiotics. Broad-spectrum cephalosporins (n = 10) were the antibiotics most frequently prescribed, followed by acylaminopenicillins (n = 7) and fluoroquinolones (n = 7). Antibiotics were administered for a mean of 6 ± 2.9 days (range 2 to 12, median 5 days) prior to the onset of cholestasis. In our cohort, 75% of the patients received TPN prior to cholestasis onset. The duration of TPN prior to cholestasis onset was very short – a mean of 5.33 ± 2.9 days (range 2 to 13, median 5 days).

**Table 3:** **Mean and peak doses of vasopressors/inotropes used**

**Doses of vasopressors/ inotropes used**								
**Mean doses μg/kg/minute**					**Peak doses μg/kg/minute**			
**Epinephrine**	**Norepinephrine**	**Dopamine**	**Dobutamine**	**Patient**	**Epinephrine**	**Norepinephrine**	**Dopamine**	**Dobutamine**
n	0.07	n	2.86	No.1	n	0.15	n	5.13
						(for 2 h)		(for 2 h)
n	n	n	2.49	No.2	n	n	n	2.49
								(for 4.5 h)
n	0.02	n	n	No.3	n	0.02	n	n
						(for 6.3 h)		
n	0.07	n	n	No.4	n	0.08	n	n
						(for 20 h)		
n	0.03	n	3.97	No.5	b	0.04	n	6.66
						(for 13 h)		(for 34 h)
n	0.05	n	2.38	No.6	n	0.12	n	2.38
						(for 24 h)		(for 12 h)
n	0.05	n	n	No.7	n	0.1	n	n
						(for 3 h)		
0.13	0.17	n	4.15	No.8	0.22	0.25	n	4.39
					(for 11 h)	(for 1.5 h)		(for 33 h)
n	0.08	n	n	No.9	n	0.23	n	n
						(for 8 h)		
b	0.06	n	n	No.10	b	0.14	n	n
						(for 0.5 h)		
0.11	n	n	3.79	No.11	0.39	n	n	7.35
					(for 1.5 h)			(for 11 h)
n	0.06	n	2.28	No.12	n	0.42	n	5.21
						(for 2 h)		(for 1 h)
n	0.09	n	n	No.13	n	0.2	n	n
						(for 6 h)		
0.03	0.02	3.1	n	No.14	0.06	0.05	3.7	n
					(for 3 h)	(for 5 h)	(for 3 h)	
n	0.03	n	n	No.15	n	0.04	n	n
						(for 46 h)		
0.08	n	1.38	2.96	No.16	0.15	n	1.38	6.4
					(for 3 h)		(for 1.5 h)	(for 10 h)
4/16	13/16	2/16	8/16		4/16	13/16	2/16	8/16

b, bolus; h, hours; n, none. (in brackets: duration of peak dose application in hours).

## Discussion

Besides critical illness polyneuropathy, secondary sclerosing cholangitis is one of the complications of severe critical illness and its successful management. SSC-CIP is an important cause of unexplained acute liver failure in ICU patients and contributes to poor outcome. In SSC-CIP, critical illness or its intensive care management can convert healthy bile ducts into severely destructed ducts within only a few days. This bile duct damage is initiated within the first seven days of ICU admission. A number of risk factors are under discussion, including shock/arterial hypotension, high-dose vasopressor therapy, sepsis, total parenteral nutrition and ventilation strategies with PEEP >10 cm H_2_O.

However, the question remains, which of these risk factors play a role as drivers of bile duct damage in SSC-CIP? Following the Hill criteria, the potential risk factors should be:

1. Present prior to the onset of cholestasis (temporality),
2. Common to all patients with this problem (consistency), and
3. Biologically plausible, i.e. capable of driving the pathogenesis to **“toxic bile”** or to **“ischemic cholangiopathy”** (plausibility) [6].

Based on a review of the current literature, our study is the first large case series to specifically investigate the suspected causes of SSC-CIP and their biological plausibility. Further larger prospective studies are needed to settle these issues. Proof of temporality, consistency and plausibility is a *sine qua non* condition for investigation of the causal mechanisms using other study designs. Our data provide the basis for future research. We started from the hypothesis that only those triggers of SSC-CIP that are consistently observed in all SSC-CIP patients should be seriously considered.

Bile duct destruction in SSC-CIP appears to be the consequence of a single event, during which a large number of cholangiocytes are damaged directly and irreversibly. Direct damage to the cholangiocytes can be induced by either **ischemia/hypoxia** or by the detergent properties of so-called **‘toxic bile’. Ischemia** and **‘toxic bile’** are conceivable as two underlying pathophysiological mechanisms of SSC-CIP.

### Concept of ischemic cholangiopathy

In contrast to hepatocytes, which have a dual blood supply, the biliary epithelium is supplied exclusively via the hepatic arteries. In consequence, the biliary epithelium is much more susceptible to ischemia than hepatocytes. Prior to the late 1960s, ischemic cholangiopathy was virtually unknown, and our clinical information on ischemia-induced bile duct lesions is based almost solely on experience with liver transplantation [7]. Early after liver transplantation, interruption of the arterial blood supply (for example, by hepatic artery thrombosis), results in bile duct necrosis. Further support for the concept of ischemic cholangiopathy came from observations in the 1980s, showing that the administration of floxuridine in the hepatic artery results in bile duct necrosis and sclerosing cholangitis [8,9]. Ischemic necrosis of biliary epithelium can occur when the blood supply to the bile ducts is disturbed at the level of either the macro-circulation (that is, the branches of the hepatic artery), or of the micro-circulation (that is, the vascular network exclusively supplying the bile ducts, the peribiliary plexus (PBP)).

### Concept of ‘toxic bile’

In the absence of protective mechanisms, the detergent properties of hydrophobic bile acids would immediately destroy the lipid cellular membrane of cholangiocytes. One important protective mechanism is hepatocellular secretion of phospholipids (via the transporter phospholipid export pump MDR3), which form protective mixed micelles with bile acids. In addition, the biliary secretion of HCO_3_^−^ (via the transporter anion exchanger 2 (AE2)) results in the formation of a protective alkaline HCO_3_^−^-film on the apical cholangiocyte membrane; this is assumed to be part of the defence strategy [10]. A finely-tuned balance between bile acids and protective mechanisms is imperative for the integrity of the bile duct epithelia. Thus, disturbance of this equilibrium results in severe damage to the biliary epithelium and leads to sclerosing cholangitis [11-14]. Such an imbalance can result from alterations of the hepatobiliary transporter system.

### Drivers of the concept of ischemic cholangiopathy in our patients

In our SSC patients, several factors in the intensive care setting were capable of compromising the arterial blood supply to the bile ducts. These include states of shock with decreased MAP, the use of catecholamines, mechanical ventilation, ventilation strategies with high PEEP, paO_2_/FiO_2_ < 150 mmHg and disturbances of the microcirculation.

#### Macrocirculatory disturbances as drivers of ischemic cholangiopathy

In the case of an incipient circulatory collapse with arterial hypotension, deterioration of the hepatic perfusion with subsequent alterations of the biliary blood supply is to be expected because the perfusion of the liver can be maintained by autoregulation only to a limited extent [15]. All of our 16 patients initially survived an episode of severe haemodynamic instability with a decrease in MAP to <65 mmHg**.** The later this hypotensive episode occurred during the treatment course, the later cholestasis appeared. Shock states with decreased arterial pressure are common in ICU patients – according to reports in the literature up to 58% of ICU patients are affected [16]. In comparison, the rate of 100% in our series was very high, supporting a possible role of severe hypotension in the initiation of the pathogenesis. When fluid administration fails to restore adequate systemic pressure in such a clinical situation with a critical blood pressure decrease, treatment with vasoactive agents is initiated. Therefore, all 16 of our patients required vasopressors and/or inotropes. However, since the increase in systemic blood pressure does not always correlate with improved hepatosplanchnic perfusion, prolonged liver ischemia can be masked**.** Commonly used agents, such as epinephrine, norepinephrine, dopamine and dobutamine, all increase systemic blood pressure, but have different effects on hepatosplanchnic blood flow. Dopamine not only increases the mesenteric blood flow, but apparently also has a positive effect on liver perfusion [17]. Only 2 of our 16 patients received dopamine. In contrast, epinephrine and norepinephrine have a dose-dependent negative effect on the perfusion of visceral organs. On account of its vasoconstrictive properties, norepinephrine has the potential to decrease splanchnic blood flow. Epinephrine, in particular, is known for its disadvantageous effect on splanchnic perfusion in septic patients [18,19]. In our series, norepinephrine was the most frequently used vasopressor (13/16 patients), and epinephrine was used only rarely (4/16 patients); one patient received neither drug. The maximum dose of epinephrine or norepinephrine exceeded 0.2 μg · kg^−1^ · min^−1^ in the minority of patients (5/16). The majority of patients received catecholamines in the medium or low-dose range. Neither in the epinephrine (E) nor in the norepinephrine (NE) group was the mean continuously administered dose (MCAD) within the high-dose range (>0.2 μg · kg^−1^ · min^−1^); in both groups, the MCAD was mostly in the range below 0.1 μg · kg^−1^ · min^−1^ (E: n = 2/4, NE: n = 12/13). Overall, our results do not support the earlier assumption that high-dose catecholamines play a decisive role in the pathogenesis of SSC-CIP.

#### Microcirculatory disturbances as drivers of ischemic cholangiopathy

As is known from experimental models, ischemic bile duct damage increases as the size of occluded arteries decreases [20-22]. This indicates that microcirculatory disturbances involving the peribiliary plexus are of particular clinical significance. The question as to why SSC-CIP occurs much more often in surgical patients has yet to be answered. Two-thirds of all published cases of SSC-CIP were patients with trauma, burns or major surgery. Also in our group, 13 of the 16 cases had surgery-requiring primary diseases (Table 1). Polytrauma or extensive surgery is usually accompanied by extensive tissue damage. Clinical studies show that this tissue damage can be an initiator of a hypercoagulable state [23-28]. This might well contribute to occlusion of the peribiliary plexus**.** Ischemic bile duct damage has frequently been found in a number of conditions known to be associated with hypercoagulopathy, such as the antiphospholipid syndrome, paroxysmal nocturnal haemoglobinuria, Schönlein-Henoch purpura or systemic lupus [29-33]. Other important factors affecting the microcirculation are increased blood viscosity and red cell aggregation. Trauma, burns and major surgery are all conditions that may increase plasma viscosity. Furthermore, extensive muscle destruction, electric power accidents and post-ischemic syndrome are often associated with a marked release of myoglobin (crush syndrome) which can result in an increase in blood viscosity, as shown in animal experiments [34]. However, the infusion of colloidal solutions, such as hydroxyethyl starch (HES), can also increase whole blood viscosity [35,36]. HES is also one of the components of the University of Wisconsin (UW) solution, used in the preservation of donor livers for transplantation. Unfavourable effects of this solution on blood flow through the peribiliary plexus have been reported in clinical studies, and are supported by experimental data [37,38]. In recent years, it became clear that there are risks of RBC transfusion related to RBC storage effects. Stored RBCs adhere to the endothelium or lose their deformability, resulting in obstruction of the microcirculation. Increased mortality rates associated with RBC transfusion have been reported in cardiac surgery patients and after liver transplantation [39-41]. Of our patients, 81% (13/16) received blood transfusions prior to onset of cholestasis. This is a very high proportion compared to the percentage (45%) generally reported in ICU patients [42,43]. Increased blood viscosity caused by colloids or myoglobin release, RBC transfusion and hypercoagulable states are among the factors determining microcirculatory blood flow through the PBP. All 16 patients had at least one of these factors – often several simultaneously – prior to the onset of cholestasis. These observations suggest that apart from severe hypotension, additional microcirculatory disturbances may be involved in the pathogenesis of SSC. Possibly, severe hypotension may have bile duct implications only when it occurs in combination with additional microcirculatory disturbances.

#### Mechanical ventilation as driver of ischemic cholangiopathy

Experimental studies indicate that mechanical ventilation with high PEEP for lung-protective purposes has negative effects on the microcirculation in the gastrointestinal tract [44]. Based on these studies, it was suspected, that, in particular, a high PEEP contributes to biliary ischemia, and that patients with a low oxygenation index are at risk of developing SSC-CIP [2]. Our results demonstrate that the development of SSC-CIP is not conclusively associated with a PEEP >10 cm H_2_O or a PaO_2_/FiO_2_ < 150 mmHg. However, this does not exclude the possibility that mechanical ventilation itself might be of significance, since all cases of SSC-CIP described in the literature as well as our own 16 patients, received mechanical ventilation.

### Drivers of the ‘toxic bile concept’ in our patients

All of our patients were exposed to several risk factors potentially leading to biliary injury via ‘toxic bile’ effects mediated through alterations of the hepatobiliary transporters. These risk factors include SIRS/sepsis, ischemia, high-dose propofol, antimicrobial agents and total parenteral nutrition.

#### SIRS/sepsis could be a driver of ‘toxic bile*’* in SSC-CIP

Pro-inflammatory cytokines such as TNF-α, IL-1 or IL-6 down-regulate the function and/or expression of hepatobiliary transporters. Animal models indicate, for example, that the canalicular bilirubin-conjugate-export pump (MRP2) is sensitive to cytokines and that the mRNA of the bile salt export pump (BSEP) is down-regulated under the influence of inflammatory mediators [45-47]. These data have been confirmed in human hepatocytes [48]. However, data regarding the phospholipid export pump (MDR3) are not conclusive, but some suggest a decrease in its hepatic expression in experimental inflammation models [47,49]. At the biliary transporter level**,** pro-inflammatory cytokines inhibit the activity of the anion exchanger AE2 [50]. In the clinical setting, the development of SIRS reflects cytokine release. Such cases of SIRS developed in all of our patients prior to cholestasis. SIRS is a common finding in patients in the ICU**,** usually occurring in 68% of the cases [51]. By comparison, the 100% incidence of SIRS in our group was exceptionally high. Therefore, if *plausibility, temporality* and *consistency* (Hill criteria) are given, SIRS could play a role in triggering SSC-CIP. Our patients exhibited signs of SIRS within 4.4 ± 2.9 days of ICU admission (range 2 to 11, median 3 days). However, up to this point, a pathogen was isolated in only 10/16 patients. Thus, infections do not adequately explain the development of SIRS in our patients. The release of pro-inflammatory cytokines is not limited to bacterial infections, but is also triggered by trauma, severe burns, cardiac surgery and other major surgery [52]. Moreover, prolonged hypotension or shock can also induce SIRS via generalised tissue ischemia [53]. In 14 of our 16 patients SIRS was preceded by severe hypotension, which could explain the non-infectious SIRS in our series.

#### Ischemia/hypoxia could be a driver of ‘toxic bile*’* in SSC-CIP

All 16 patients in our study had experienced an episode of severe hypotension. Experimental data suggest that ischemia/hypoxia can also down*-*regulate hepatobiliary transporters [54].

#### High*-*dose propofol seems not to be a driver of ‘toxic bile’ in SSC-CIP

Propofol, which is commonly used for sedation in ICU patients, is thought to influence hepatobiliary transporters via the release of cytokines. While high-dose propofol (5 to 10 mg kg^−1^ h^−1^) increases serum levels of TNF-α and IL-10 after haemorrhagic shock, low-dose propofol (1 mg kg^−1^ h^−1^) decreases them [55]. Since only one of our patients received high-dose propofol, this mechanism played no key role in our patients.

#### Total parenteral nutrition seems not to be a driver of ‘toxic bile in SSC-CIP

Clinical studies have shown that prolonged parenteral nutrition can trigger cholestasis. In a mouse model the expression and function of the canalicular phospholipid flippase (Mdr2) significantly decreased under the influence of TPN [56]. However, 25% of our patients did not receive TPN prior to the onset of cholestasis. In the 75% with prior TPN, the average TPN duration was very short (five days). In contrast, TPN-induced cholestasis usually occurred only after two to three weeks of therapy. Likewise, the results of a multicentre study by Grau *et al*. showed no impairment of liver function within the first six days of TPN [57]. In the light of these findings we do not attribute any major role to TPN in the pathogenesis of SSC-CIP in our patients.

#### Antimicrobial agents/antibiotics seem not to be a driver of ‘toxic bile*’* in SSC-CIP

Inhibitory effects of antimicrobial substances, such as rifampicin and fusidate, on the function of hepatobiliary transporters have been reported [58,59]. In a recent study by Yosikado *et al*. itraconazole was shown to inhibit MDR3-mediated efflux of phospholipids *in vitro* and led to itraconazole-induced cholestatic liver injury *in vivo* [60]. Although all of our patients received antibiotic therapy, the spectrum of the substances was so heterogeneous that a common pathogenetic pathway appears improbable.

### Other possible causes

#### Anaesthetic agents could be drivers for SSC*-*CIP

A number of cases of ketamine-induced cholangiopathies were recently reported [61-63]. A large proportion of our patients (15/16) received ketamine prior to the onset of cholestasis, and it cannot be excluded that this had an unfavourable effect on the course of the illness.

### Prevalence of SSC-CIP

The true prevalence of SSC-CIP is hard to estimate since the disease still is not well known and is thus under-diagnosed in many areas. Systematic reporting of diagnosed cases is also lacking. Unfortunately, our method of data collection is not capable of calculating valid SSC-CIP prevalence rates. Of all liver transplantations in our centre, 0.61% (16/2633) were due to SSC-CIP. Similar rates have been observed, for example, in patients with hemochromatosis (0.7% of all liver transplantations at our centre). This allows the assumption that SSC-CIP is – similar to hemochromatosis– rare in the general population. But this is not the case with critically ill patients. In our experience, SSC-CIP seems to occur more frequently at trauma or cardiac surgery ICUs than in the internal medicine ICU. We found 5 cases of SSC-CIP in a total of 9951 ICU cases at one trauma ICU (1 SSC-CIP/1,990 ICU-cases). It must be assumed that these figures underestimate the actual number of cases. It is known that 50% of ICU patients die of SSC-CIP at an early stage of intensive care, before the diagnosis is established [64]. A better understanding of SSC-CIP and registers for systematic reporting of the disease could clarify the epidemiology of secondary sclerosing cholangitis in critically ill patients in the future.

## Conclusions

With regard to temporality, consistency and plausibility, the following potential triggers might contribute to the development of SSC-CIP: severe hypotension, microcirculatory disturbances and SIRS/sepsis. Further clinical and experimental studies can build on and consolidate these results. Severe hypotension, microcirculatory disturbances and SIRS/sepsis may act in concert to induce damage to the bile ducts. While severe hypotension can cause direct ischemic bile duct damage, it might also contribute indirectly to destruction by altering hepatobiliary transporters. Although both severe hypotension and SIRS/sepsis can individually affect hepatobiliary transporters, their effects might possibly be potentiated in combination. In summary, it appears that severe hypotension/shock is the first step in the pathogenesis of SSC-CIP. While more recent investigations show that hypoxic hepatitis can no longer be termed as ‘shock liver’, the development of SSC-CIP could represent the actual response of the liver to shock.

## Key messages

- SSC-CIP is triggered by severe haemodynamic instability with a prolonged decrease in mean arterial pressure (MAP).
- Apparently, this decrease in arterial pressure contributes to critical perfusion of the peribiliary plexus only if additional microcirculatory disturbances (colloids, RBCs, hypercoagulable state, increased plasma viscosity) exist.
- Non-infectious SIRS could be an additional cause of SSC-CIP.
- Neither high-dose catecholamines nor total parenteral nutrition (TPN) plays a decisive role in triggering SSC-CIP.

## Abbreviations

AE2: anion exchanger 2
ALP: alkaline phosphatase
CK: creatine kinase
COPD: chronic obstructive pulmonary disease
ERC: endoscopic retrograde cholangiography
GGT: gamma-glutamyl transpeptidase
HES: hydroxyethyl starch
ICU: intensive care unit
IL-1: −6 or 10, interleukin- 1, −6 or 10
MAP: mean arterial pressure
Mdr2: multidrug resistance protein 2
MDR3: multidrug resistance protein 3, phospholipid export pump
MRP2: multidrug resistance- associated protein 2, conjugate export pump
PBP: peribiliary plexus
PEEP: positive end-expiratory pressure
PSC: primary sclerosing cholangitis
RBC: red blood cells
SSC-CIP: secondary sclerosing cholangitis in critically ill patients
SIRS: systemic inflammatory response syndrome
TNF-α: tumor necrosis factor α
TPN: total parenteral nutrition
